# Axonal Transport Impairment in Chemotherapy-Induced Peripheral Neuropathy

**DOI:** 10.3390/toxics3030322

**Published:** 2015-08-07

**Authors:** Gabriella Nicolini, Marianna Monfrini, Arianna Scuteri

**Affiliations:** 1 Experimental Neurology Unit and Milan Center for Neuroscience, Department of Surgery and Translational Medicine, University of Milano-Bicocca, via Cadore 48, 20900 Monza, Italy; E-Mails: gabriella.nicolini@unimib.it (G.N.); m.monfrini1@campus.unimib.it (M.M.); 2 PhD Neuroscience Program, University of Milano-Bicocca, via Cadore 48, 20900 Monza, Italy

**Keywords:** axonal transport, neurotoxicity, peripheral neuropathy

## Abstract

Chemotherapy-Induced Peripheral Neuropathy (CIPN) is a dose-limiting side effect of several antineoplastic drugs which significantly reduces patients’ quality of life. Although different molecular mechanisms have been investigated, CIPN pathobiology has not been clarified yet. It has largely been recognized that Dorsal Root Ganglia are the main targets of chemotherapy and that the longest nerves are the most damaged, together with fast axonal transport. Indeed, this bidirectional cargo-specific transport has a pivotal role in neuronal function and its impairment is involved in several neurodegenerative and neurodevelopmental diseases. Literature data demonstrate that, despite different mechanisms of action, all antineoplastic agents impair the axonal trafficking to some extent and the severity of the neuropathy correlates with the degree of damage on this bidirectional transport. In this paper, we will examine the effect of the main old and new chemotherapeutic drug categories on axonal transport, with the aim of clarifying their potential mechanisms of action, and, if possible, of identifying neuroprotective strategies, based on the knowledge of the alterations induced by each drugs.

## 1. Axonal Transport

Axonal transport is a key process of neuronal functionality, due to the peculiar feature of neurons, where the soma can be a long distance from the distal cellular parts of the neurites. At the soma level all the fundamental processes occur, such as DNA replication, transcription, and overall the mRNA translation into proteins, which afterwards must be driven to and along axons to exploit their function. Indeed, the function of axonal transport is to drive proteins and organelles to the distal parts of the cells, thus connecting the heart of the cell with the periphery [1]. However, the description of axonal transport as a simple refilling method to guarantee the correct axonal composition, even if pivotal, will be much too restrictive, since the communication from periphery to soma is also extremely important. Axonal transport is composed of two distinct mechanisms, a fast axonal and a slow component [1]. The fast mechanism is a vesicular transport based on microtubules. It can be anterograde, when directed from soma to axons, and in this case it uses kinesin as motor protein, or retrograde, going from axons to soma, and using dynein as motor protein. To guarantee the correct progress of axonal transport, all the involved molecules must be precisely regulated, and the regulation may occur by post-translational modifications of motor proteins [2]. Moreover, the microtubules are also pivotal to axonal transport, and as well as the motor proteins, they can be precisely regulated by the involvement of Microtubule-Associated Proteins (MAPs). It has been extensively demonstrated that changes in microtubule dynamics (and therefore in axonal transport) are largely due to post-translational modifications of both motor proteins and MAPs (*i.e.*, tau) [3].

**Table 1:** Summary of drug mechanisms of axonal toxicity.

Drug	Mechanism	Citation
*Taxanes*	Perturbation of microtubules dynamics and stability	[4,5]
	Reduction of kinesin-dependent transport	
*Vinca Alkaloids*	Destabilization of lateral interactions between protofilaments	[5,6]
	Reduction of kinesin-dependent transport	
*Eribuline*	Depolymerization of microtubule tracks	[5]
	Reduction of kinesin-dependent transport	
*Epothilones*	Reduction of kinesin-dependent transport	[5]
*Cisplatin*	Damage to kinesin and dynein by adduct formation	[7]
*Oxaliplatin*	Alteration of Na^+^ channels kinetics in sensory neurons	[8]
*Thalidomide*	Inhibitive action on NF-κB that interferes with NGF activity	[9]
*Bortezomib*	Blockage of correct turnover of axonal proteins	[10]

Differently from the fast axonal transport, the slow mechanism can only be anterograde, and it mainly concerns cytoskeletal elements [11]. For neuron structural integrity, the anterograde transport is important, but neurons are also characterized by the ability to respond to external stimuli, in particular to neurotrophic factors, selecting the neurons which have to survive and determining their function. To this aim, the neurotrophic factor signaling pathway exploits the retrograde axonal transport [1]. Since the axonal transport is essential for neuronal survival, the alteration of its functionality has devastating effects. For this reason, any drug able to interfere with this process, both directly and indirectly by acting on its post-translational regulation, can virtually have a neurotoxic action (Table 1). Confirming this hypothesis, the axonal transport is one of the targets of a class of drugs, the chemotherapeutic agents, which frequently share the induction of a dose-limiting peripheral neuropathy, known in literature as Chemotherapy-Induced Peripheral Neuropathy (CIPN) [12,13].

## 2. Chemotherapy-Induced Peripheral Neuropathy (CIPN)

CIPN is a side effect that occurs in a large percentage (30%–80%) of cancer patients, very often representing the dose-limiting side effect [14]. CIPN is generally a sensory dose-dependent neuropathy characterized by paresthesia, sensory loss, dysesthesia, numbness and sometimes by neuropathic pain [15,16]. Less frequently CIPN shows motor or autonomic signs. It has been reported in antitubulin agents, platinum derivatives, proteasome inhibitors and Thalidomide treatment [17]. It is noteworthy that even inside each class of drugs, CIPN features and severity could be different [18]. CIPN includes both neuronopathy, axonopathy and myelinopathy [13,19,20]. The exact CIPN pathophysiology has not been clarified yet, even if several possible mechanisms have been identified [13]. DNA damage, mitochondrial derangement, Reactive Oxygen Species (ROS) over-production, inflammation, glutamate toxicity, proteasome or channel dysfunction and axonal transport defects are mechanisms reported to be correlated to peripheral neuropathy induced by different classes of antineoplastic drugs [12,18]. Dorsal Root Ganglia (DRG) are the main target of antineoplastic drug. DRG, in fact, vascularized by fenestrated endothelial cells lacking tight junction, are unprotected by a barrier comparable to the Blood Brain Barrier (BBB) and are exposed to different classes of low and high molecular weight drugs [21]. These data explain why CIPN is predominantly a sensory pathology. Moreover, although in all neurons axon structure and functions are strictly dependent on perikaryon organelles activity, it is widely demonstrated that the longest nerves are generally the most damaged (extensively reviewed in reference [17,22]) and CIPN is usually characterized by a dying-back neuropathy [13]. Epidemiological data suggest that diabetes mellitus might be a risk factor associated with CIPN development [23] and in the last few years a growing number of pharmacogenomic studies have reported genes and single nucleotide polymorphism (SNPs) correlated to CIPN development. In particular, polymorphisms have been demonstrated in DNA repair, growth factors, metal transporter, chemotherapy resistance and glutathione metabolism genes [24,25,26,27], but no data have been reported to clarify the greater vulnerability of longest fibers.

To date, despite important improvements in cancer treatment and many preclinical and clinical trials, no effective strategy for neuroprotection has been developed [17,28].

## 3. Antitubulin Agents

Microtubules are essential components of eukaryotic cells cytoskeleton. The basic units of microtubules are αβ-tubulin heterodimers that polymerizing produce 13 protofilaments organized to form the microtubule cylinder. In neuronal cells, besides their central role in mitotic spindle and centrioles formation, microtubules form the tracks on which bidirectional fast axonal transport is based [1]. The right balance of treadmilling and dynamic instability is crucial for microtubule functions. Consequently, antitubulin agents, both improving polymerization and/or depolymerization, alter cell mitosis and axonal transport. Considering cancer cells high mitotic index, antitubulin agents represent a widely used class of drugs, though characterized by a very high neurotoxicity.

## 4. Taxanes

Taxanes are plant derived, and Paclitaxel (a natural compound) and Docetaxel (a semisynthetic compound) are the most effective members of this antineoplastic family in the treatment of several solid tumors such as ovarian, lung, breast, prostate and head and neck cancer [29].

It has been demonstrated that taxanes antineoplastic effect is due to their ability to interfere with mitotic spindle formation in tumor cells [4]. However, this mode of action cannot be the responsible for well-known taxanes-induced peripheral neurotoxicity [30]. Paclitaxel-induced peripheral neuropathy is characterized by a predominant sensory neuropathy manifesting with bilateral paresthesia, numbness, tingling and burning pain. Moreover, deep tendon reflex loss is an early symptom of Paclitaxel neurotoxicity [19,31]. Docetaxel causes symptoms similar to those of Paclitaxel but generally they are less severe, probably due to the lower dose used [32].

The high binding affinity of taxanes to β-tubulin, in addition to blocking mitosis, can perturb microtubule dynamics and stability, inducing microtubule stabilization and determining neuronal damage by different mechanisms. Several experimental models have demonstrated that taxanes induce microtubule polymerization and inhibit depolymerization at the minus end of microtubule [4,33]. Along the microtubule length, Paclitaxel binds to β-tubulin inside the microtubules scaffold in a site including M-loop (β281–293) [34]. The binding stabilizes GDP-bound αβ-tubulin heterodimers [35] in a specific and reversible manner. In addition to stabilizing each protofilament structure, Paclitaxel binding also increases the interaction between neighboring protofilaments promoting over stable microtubule bundles formation [34,36] and altering the number of protofilaments into each microtubule [37,38].

An *in vivo* rat model has demonstrated that DRG are Paclitaxel main site of accumulation [21] and *in vitro* experiments have investigated Paclitaxel toxicity in organotypic DRG and in sensitive DRG primary culture [39]. Several studies have investigated the effect of Paclitaxel-induced polymerization on axonal transport in both *in vitro* and *in vivo* experiments. Theiss and Meller [40] have shown that *in vitro* 10 µM Paclitaxel decreases anterograde axonal transport of horseradish (HPR) peroxidase in chick DRG neurons. Simultaneously, in axons abnormal aggregation of microtubules appears. Paclitaxel (200 µM) impaired anterograde and retrograde transports have also been demonstrated by Nakata and Yorifujj [41] in Paclitaxel-bathed rat sciatic nerve using rhodamine-labeled wheat germ agglutinin (WGA-rhodamine). According to these data, but using lower concentrations of Paclitaxel (1, 10, 100 nM) more comparable to clinical doses, Goshima *et al.* [42] have demonstrated that Paclitaxel is able to reduce both the anterograde and the retrograde transport of chloro-methylbenzamido dialkylcarbocyanine (CM-Dil)-labeled organelles in chick DRG neurons. Shemesh and Spira [43] have demonstrated that Paclitaxel concentrations (10, 100 nM) similar to those used by Goshima *et al.* [42] are able to interfere with axonal transport also in an *in vitro* model of neurons isolated from buccal ganglia of the marine mollusk Aplysia Californica. Retrograde axonal transport impairment in this *in vitro* non mammalian model resulted irreversible and associated with microtubular polar reconfiguration that determines a new chaotic steady state. Moreover, 10 and 100 nM Paclitaxel also reduced the bidirectional transport of mitochondria. Shemesh and Spira [43] have suggested several hypotheses about how the polar reconfiguration may affect transport, but at the moment none of these has been demonstrated and no data regarding microtubules polar reconfiguration in mammalian peripheral neurons models have been published. However, it is noteworthy that Aplysia Californica and human tubulin share 96% of sequences [44] and this suggests that the mechanisms through which Paclitaxel acts on microtubule could be the same. In a different model, using both vesicle transport/squid axoplasma and microtubule gliding assay, La Pointe *et al.* [5] have shown that the interaction of Paclitaxel with microtubules slightly reduces the anterograde transport (12% and 17%, respectively at 1 µM and 10 µM), but it is not able to alter the retrograde transport. Moreover, in the same paper the authors have demonstrated that Paclitaxel is able to reduce kinesin-dependent transport velocities in isolated axoplasm, but it does not perturb the kinesin-1 activity in microtubule gliding assay. These data suggest that Paclitaxel action could target non-motor MAPs or regulatory proteins of motor proteins (*i.e*., kinases) that were not present in gliding assay. Among these latter there are regulatory molecules, such as kinases and phophatases that inducing post-translational modifications of kinesin or dynein influence their ability to bind microtubule and consequently affect the axonal transport [45,46,47]. Such a regulation has been already demonstrated in several neurodegenerative diseases [48] and could not be excluded in taxanes neurotoxicity. In fact, phosphorylation is a typical post-translational modification after taxanes treatment [49]. In non-cancer cells, Figueroa-Masot *et al.* [50] have highlighted activation of a sub-pool of *N*-terminal c-Jun protein kinase (JNK) in nucleus of cortical neurons and Nicolini *et al.* [51] have demonstrated a sustained phosphorylation of JNK in human neuroblastoma cell line after exposure to Paclitaxel. Moreover, it is known that JNK is able to negatively regulate fast axonal transport perturbing the motor protein kinesin activity [52,53].

Furthermore, taxanes binding sites are located on the inner surface of microtubule and consequently drug binding cannot directly affect the motor MAPs interaction with microtubule. According to this hypothesis taxanes binding should induce conformational change of microtubule that, in turn, affects the axonal transport. Hammond *et al.* [54] have demonstrated that Paclitaxel, inducing tubulin post-translational modification (acetylation, detyrosination and polyglutamination), is able to perturb the motor protein kinesin-1 ability to interact with tubulin. Among non-motor MAPs, a key role in neural microtubule development and regulation is played by tau protein [55,56]. Tau protein induces bundling and stabilization of axonal microtubules. Several studies both *in vitro* and *in vivo* have investigated the effect of tau protein overexpression on axonal transport, demonstrating impairment of cargo transport along axon [57,58,59]. Tau protein is able to bind microtubules in two sites, one of which partially coincides with that for Paclitaxel [60,61]. Treatment with Paclitaxel may then modify the binding of tau protein to microtubules thus affecting axonal transport. Samsonov *et al.* [62] have demonstrated that Paclitaxel treatment reduces tau binding to microtubule in Xenopus neurons. On the other hand, Black [63] in dissociated cultures of rat sympathetic neurons treated with Paclitaxel has not found decrease in tau protein interaction rate with microtubule. Theiss and Meller [40] have shown that Paclitaxel restricts tau protein to the soma in chick embryo DRG neurons.

## 5. Vinca-Alkaloids

Vinca alkaloids are derived from Catharanthus roseus plant (formerly, vinca rosea). This group of chemotherapeutic drugs includes both natural alkaloids, such as Vincristine and Vinblastine, and semi-synthetic ones, such as Vinorelbine, Vindesine and Vinflunine. These chemotherapeutic agents have a broad spectrum of activity against hematologic and lymphatic malignancies, as well as solid tumors such as ovarian, testicular, brain, non-small lung cell tumors and sarcomas [17].

Vinca alkaloids share the antineoplastic effect, based on inhibitory effect on microtubule assembly, while differ in antitumoral spectra and toxicity. Vinca alkaloids form a stable complex with β-tubulin, binding its GTPase domain so that GTP hydrolysis is inhibited and as a consequence β-tubulin is not able to polymerize into microtubules. Despite apparent minimal structural differences among Vinca alkaloids derivatives, they induce significantly dissimilar conformational changes of β-tubulin, due to their different affinity for β-tubulin, (Vincristine > Vinblastine > Vinorelbine > Vinoflunine), and this could explain the distinct neurotoxic profiles: Vincristine is the most effective drug but it is also the most neurotoxic [6,17]. Vincristine-induced peripheral neuropathy is characterized by disturbance in sensory, motor and autonomic functions [18], and the severity of symptoms depends on several factors: (i) patient age [64]; (ii) concomitant use of other neurotoxic modalities, such as radiation therapy and chemotherapeutic agents [65]; (iii) dosage regimen [66]; (iv) method of calculation of total dose [67].

The mechanism that underlies Vinca–alkaloid-induced neuropathy remains still unclear, but many possible causes could be involved, such as changes in gene expression, membrane excitability, inflammation and also axonal transport disruption [16,68,69]. The Vincristine inhibitory effect on β-tubulin polymerization leads to a severe alteration in axonal microtubules, which entails axonal swelling in myelinated and unmyelinated fibers [70], due to a progressive accumulation of axoplasmic organelles and vesicles [71] and damage to nervous fibers. Vincristine also induces chronic neuropathic pain, because C-fibers nociceptors enhance their responsiveness [72]. Vinblastine induces a less severe peripheral neuropathy in comparison with Vincristine, but a hematological toxicity is observed. Vinorelbine and Vinflunine administration causes a mild sensory-motor neuropathy, which is reversible after drug discontinuation [73]. Furthermore, C-fiber dysfunction is not observed, saving patients from chronic pain.

Vincristine limitations have prompted the improvement of Vincristine formulation, and liposomal technology has been proposed; the use of Vincristine sulfate liposome is associated with a more favorable PK profile and clinical outcomes [74]. As reported for Exabepilone, Vincristine has severe inhibitory effects on fast axonal transport, both on anterograde and retrograde direction. In common with Eribulin, Vincristine suppresses microtubule dynamic instability at low concentration and promotes microtubule disassembly at high concentration [75,76,77]. Since Vincristine binds along microtubule length, the interactions between microtubules and motors (in particular kinesin-1) are affected, and even dimeric tubulin conformation is compromised [5].

## 6. Epothilones

Epothilones are anti-tubulin agents derived from the myxobacterium Sorangium cellulosum, which show high resistance to multidrug resistant (MDR) mechanisms [78] and are effective against cells that express the multidrug resistance gene MDR-1 and have acquired tubulin mutations. Among epothilones the two major fermentation products are Epothilone A and Epothilone B.

Ixabepilone (BMS-227550) is a second-generation semisynthetic analogue of Epothilone B that shows high metabolic stability, low plasma protein binding and activity especially against taxanes-resistant cell lines [79,80]. Despite Ixabepilone being a good therapeutic option for advanced or metastatic breast cancer, 72% of patients manifest neuropathic symptoms [81]. However, it is noteworthy that the incidence of its sensory neuropathy is very variable among different administration schedules [82,83].

Sagopilone (ZK-EPO) is a fully synthetic third-generation Epothilone B derivative [84]. It has been demonstrated to be active against breast cancer, non–small cell lung cancer, cholangiocarcinoma, melanoma and adrenal carcinoma [85]. Moreover, Hoffmann *et al.* [86] have demonstrated that Sagopilone is able to cross the blood-brain barrier. Although two different phase II trials failed to demonstrate Sagopilone’s efficacy against glioblastoma and brain metastasis [87,88].

Epothilones antineoplastic activity is related to their ability to bind microtubules, leading to aberrant spindle formation that determines mitotic arrest and apoptosis through Bcl-2 phosphorylation [85]. In particular, epothilones bind to tubulin in a site next to taxanes site on β-tubulin inside the microtubule and along the microtubule length. Epothilones binding determines interaction between tubulin dimers intra and inter protofilaments promoting microtubule assembly and reducing their dynamic instability [89].

Epothilones-induced peripheral neuropathy is primarily an axonal, dose-dependent, sensory distal neuropathy which is reversible. Paresthesia, dysesthesia, numbness and/or burning neuropathic pain in a stocking-glove distributions are commonly reported symptoms [83].

La Pointe *et al.* [5] have demonstrated that Ixabepilone reduces both kinesin-dependent transport velocities in isolated axoplasma and kinesin activity in microtubule gliding assay. The observation that inhibition in entire axoplasm is higher than in *in vitro* gliding assay suggests that the inhibition of Ixabepilone on axonal transport could be due to its effect on non-motor MAPs present in axoplasma.

Moreover, the greater effect of Ixabepilone on axonal transport and the more severe peripheral neuropathy with respect to those induced by Paclitaxel suggest different post-translational modification of tubulin and different effect on microtubule organization. In fact, Meurer-Grob [90] has demonstrated that epothilones (like Paclitaxel) are able to modify the microtubule protofilament number and Khrapunovich-Baine *et al.* [36] have shown that both epothilones and Paclitaxel affect the COOH-terminus of tubulin influencing the binding of motor and non-motor MAPs and in particular the regulation of kinesin-1 activity.

## 7. Eribulin

Eribulin mesylate (E7389) is an anticancer drug belonging to the anti-microtubule agents and successfully administered in patients with advanced metastatic breast cancer [91,92]. Moreover, Eribulin is active also against Cisplatin-resistant ovarian cancer [93]. Structurally it is a simplified macrocyclic, ketone analogue of halicondrin B [94]. Its antitumoral efficacy, demonstrated in *in vitro* experiments on several cell lines (U937, Jurkat, HL-60, and HeLa cells) and in four different xenograft models (breast, colon, ovarian cancer and melanoma), is due to irreversible mitotic blockade, related to sustained Bcl-2 phosphorylation [95,96]. Eribulin mesylate binds to the plus end of microtubules (affecting polymerization), but its binding does not influence microtubule depolymeralization [77,97]. Furthermore, after Eribulin mesylate treatment, abnormal mitotic spindles unable to undergo metaphase/anaphase checkpoint are detectable. Formation of mitotic spindle is due to non-functional aggregates of tubulin after Eribulin [75,98].

Regarding CIPN induced by Eribulin, low incidence (21%–26%) of severe neuropathy has been reported [97]. According to this clinical observation, La Pointe *et al.* [5], using vesicle motility assay in isolated squid axoplasma, have demonstrated that Eribulin has a mild effect on fast axonal transport. In particular, Eribulin is able to slightly reduce anterograde fast axonal transport (10% and 13% respectively at concentrations of 1 µM and 10 µM), but it does not inhibit retrograde fast axonal transport. In contrast, Eribulin is ineffective in reducing kinesin-1 activity in microtubule gliding assay. This mild effect could be due to the Eribulin binding only to the plus end and not along the entire microtubule.

Other drugs, differently from taxanes and Vinca alkaloids, which clearly affect the inner structure of axonal transport, other classes of anticancer drugs do not have the microtubule system as main target, but anyway they induce the onset of a peripheral neuropathy, simply through different mechanisms.

## 8. Platinum Compounds

Platinum compounds are a family of several antineoplastic drugs all characterized by the presence of a Platinum (Pt) atom in their chemical structure. Their common antineoplastic action is based on the formation of adducts between Pt and DNA and/or proteins, which induce the apoptotic death in replicating cells. However, also the post-mitotic neuronal cells are damaged by the drugs, in particular the DRG sensory neurons [15]. The DRG become an accumulation site for the drugs, and the target of their neurotoxic action [15]. Although similar, these drugs have different efficacy, and also show a different and in some case peculiar neurotoxic profile, so the consequences of the neurotoxic effect depend on the drug, more serious with Cisplatin and milder with Carboplatin [99].

## 9. Cisplatin

Cisplatin (*cis*-diamminedichloroplatinum(II)) is the first drug of the platinum compounds family entered in clinical practice, effectively used for the treatment of several tumors, such as ovarian, testicular, and small cell lung cancers [99]. Starting from a cumulative dose of 300 mg/m^2^ neuropathic signs appear, and over the cumulative dose of 500 mg/m^2^ a severe sensory neuropathy has been reported in a large amount of patients, with distal paresthesia and sensory ataxia [15,100]. Sometimes these symptoms progress for several months after the end of drug treatment, an event known as “coasting” [100].

Initially it has been hypothesized an axonopathy, with a “dying back” degeneration mechanism, since neurophysiological studies have evidenced a reduction of Sensory Nerve Action Potentials (SNAPs) amplitude [101]. However, several authors have demonstrated that Cisplatin induces neuronal cells degeneration through different mechanisms. Surely the formation of Pt-DNA and Pt-proteins adducts has adverse effects also for neurons, but Gill and Windebank [102] have demonstrated that these cells after Cisplatin exposure undergo to an attempt to re-enter into the cell cycle from G0 phase, and this event is the prelude that triggers the apoptotic neuronal death.

It is noteworthy that an *in vitro* study reported Cisplatin ability to directly damage the motor proteins kinesin and dynein by adduct formation, and so to directly impair the fast axonal transport [7], but the available evidence suggests that Cisplatin exposure more likely induces the onset of a neuronopathy. Therefore, axonal degeneration could be only the consequence of the anterograde transport impairment and it may also explain the Cisplatin “coasting” effect, as the latency needed by damage to reach the distal part of the axons.

## 10. Carboplatin

Carboplatin [*cis*-Diammine(1,1-cyclobutanedicarboxylato)platinum(II)] is a Cisplatin analogue frequently used as first line treatment for advanced ovarian carcinoma [103]. It is characterized by a lower neurotoxic profile, although at high doses (1600 mg/m^2^) it can induce a neuropathy very similar to that observed after Cisplatin treatment, with painful paresthesia and sensory ataxia [104].

## 11. Oxaliplatin

A one-off case is represented by Oxaliplatin [*trans*-*R*,*R*-cyclohexane-1,2-diamineoxalatoplatinum(II)], a 3rd generation drug used as first line treatment for colorectal cancer [100]. Oxaliplatin is characterized by a unique neurotoxic profile, since, besides the classical platinum-dependent chronic neurotoxicity, generally arising at a cumulative dose above 550 mg/m^2^, it also shows an acute toxicity, that typically develops within the first 2 days of treatment and manifests with cold-induced distal dysesthesia and paresthesia [100]. The chronic neuropathy is very similar to that induced by Cisplatin, being a neuronopathy with anterograde axonal transport impairment secondary to neuronal damage [105]. The acute neurotoxicity seems to mainly affect the axons, by the effect of oxalate salt on the Na^+^ channels present along the axons, thus determining axonal conductivity impairment [8,106]. Moreover, a study have also evidenced an important vacuolization of axonal mitochondrion and a marked reduction of intraepidermal nerve fiber density after Oxaliplatin exposure [107,108]. By the use of neurography, a molecular imaging technology, Schellingerhout *et al.* [109] have demonstrated that the Oxaliplatin-dependent neurotoxicity determines an impairment of the retrograde axonal transport involved in neurotrophin transport [109].

## 12. Thalidomide

The sunrise of Thalidomide story, a glutamic acid derivative, became sadly and worldwide known when, in the 1950s, it was prescribed as mild sedative and antiemetic to pregnant women, having no knowledge of the teratogenic and anti-abortive side effects, with the result of a number of children affected by serious malformations, and the consequent withdrawal of the drug from the market in the 1960s [110]. Afterwards, further studies of Thalidomide have allowed to reintroduce it in clinic for the therapy of peculiar dermatological diseases, such as leprosy, and recently the use of Thalidomide has been extended to the treatment of Multiple Myeloma, due to its anti-angiogenic and anti-inflammatory actions [111,112].

After the use of Thalidomide, with typical doses ranging from 50 mg to 300 mg/day, the onset of a dose-dependent peripheral sensory neuropathy has been reported, which symmetrically begins from the distal extremities (hands and feet), and which clinically manifests with paresthesia of the extremities [113,114,115]. Further studies confirmed that the sensory neuropathy is length-dependent [20,116]. Many studies have tried to understand the molecular mechanisms at the basis of neuropathy induction, and to identify the target of this side action. Concerning the molecular mechanisms many hypotheses have been formulated, strictly linked to the mechanisms of Thalidomide antiproliferative action [114]. The reduction of blood supply to the axons due to anti-angiogenic activity could determine their degeneration with greater damage of longest neurites. In addition, some authors focus their attention on the regulatory role of Thalidomide on cytokines signaling pathway, through the inhibition of Nuclear Factor κB (NF-κB), which has a pivotal role in Nerve Growth Factor (NGF) signaling pathway [114]. This neurotrophin is essential for neuronal survival and arborization, and therefore Thalidomide inhibitive action on NF-κB could interfere with NGF activity, thus leading to neuronal and axonal degeneration.

Thalidomide direct toxic action on neurons rather than on axons, as hypothesized by some authors, casts doubts on the main target of Thalidomide neurotoxicity [9]. The answer to this question is not purely academic, but very important when thinking about neuroprotective strategies. In fact, if the axon is the drug target, it is possible to hypothesize about reparative/regenerative therapy, but if the damage occurs on DRG neurons, it can easily be irreversible, and therefore a preventive therapy should be applied. Although *in vitro* only the neuronal degeneration has been observed, by a clinical point of view the axonal damage seems to be more relevant [17].

Since Thalidomide induces a dose-dependent neuropathy, the clinical recommendation is to use the lowest effective dose, with a careful monitoring of electrophysiological parameters which represent the first neuropathic sign, such as the reduction of SNAPs amplitude, to evaluate the drug dose reduction or even the treatment temporary or permanent withdrawal. In addition, preventive strategies such as anti-oxidant or growth factors administration are currently under evaluation [117,118], as well as the use of derivatives with a very low neurotoxic profile, such as Lenalidomide and Pomalidomide [113].

## 13. Bortezomib

Bortezomib, a boronic acid dipeptide, represents the first-in-class member of the proteasome inhibitors family, and it currently is the gold standard treatment for multiple myeloma [114]. As other antineoplastic drugs, also Bortezomib induces a dose-dependent peripheral neuropathy and patients develop a length-dependent, sensory and painful neuropathy [114,119].

Different mechanisms have been proposed to explain the neurotoxic effect, mainly ascribable to Bortezomib antiproliferative action, which is the inhibition of proteasome complex, the system by which the cell recognizes and eliminates the ubiquitinated proteins, aimed to be destroyed [119]. The inhibition of such a system induces the apoptotic death pathway cells, but it does not spare the neuronal cells. Protein aggregate accumulation can have a neurotoxic effect directly on soma neurons, but it can also block the correct turnover of axonal proteins, thus leading to axonal transport impairment and then to neuronal death. Anyway, the target of Bortezomib-induced peripheral neuropathy seems to be DRG neurons rather than axons, thus characterizing as a neuronopathy, even if sometimes the damage of Schwann cells has also been reported [120]. Some authors have reported the increased expression of TNF-alpha in DRG after Bortezomib administration, thus explaining the neurotoxic action [119]. Other authors have demonstrated a possible role of the immune system in neuropathy onset [121,122], but such a role has been recently questioned [123]. Undoubtedly Bortezomib treatment also reduces the plasmatic level of some neurotrophins, in particular of BDNF [124,125], with a consequent alteration in neuronal survival. However, Nasu and co. have demonstrated an alteration of the excitatory membrane potentials after Bortezomib administration, and therefore of the correct axonal functionality, thus focusing the attention on the axons [126]. In this way it is important to report that a direct action of Bortezomib on microtubules and on tubulin besides proteasome inhibition has been described [10,127] without evidences of a survival reduction of neither neurons nor Schwann cells [10,127,128]. It is possible that both aspects, axonopathy and neuronopathy, coexist, but probably in the clinical setting the axonopathy symptoms prevail.

Currently there are some analogs of Bortezomib under evaluation, such as Calfizomib and Marazomib, with a very mild profile of neurotoxicity [129,130].

## 14. Conclusions

Despite different mechanisms of action, the various chemotherapeutic agents share some toxic effect on axonal transport, which has the induction of a dose-limiting peripheral neuropathy as a consequence. Several neuroprotective therapies have already been tried, but with limited effectiveness [100,118,125]. The knowledge of the mechanisms underlying such neurotoxic effect has a great importance in terms of neuroprotective strategies against CIPN, since it could be hypothesized as an *ad hoc* choice of the best preventive/therapeutic approach, based on the drug and on its specific alterations to axonal transport. From this perspective, preventive strategies could partner the action of chemotherapeutic drugs targeting the DRG, while regenerative/reparative approaches could also follow the anticancer treatments. Since the onset of the peripheral neuropathy is often the main dose-limiting side effect of chemotherapy, the chance to limit it represents an huge goal to maximize the antineoplastic drug effect (and patient’s quality of life), without the need to reduce their dose or even to stop the therapy. The knowledge of the mechanisms underlying such neurotoxic effect could be helpful to identify neuroprotective strategies, in order to treat or even to prevent the neuropathy onset, and in this way to maximize the antineoplastic drug effect.
